# Modulator of Apoptosis 1 (MOAP-1) Is a Tumor Suppressor Protein Linked to the RASSF1A Protein*

**DOI:** 10.1074/jbc.M115.648345

**Published:** 2015-08-12

**Authors:** Jennifer Law, Mohamed Salla, Alaa Zare, Yoke Wong, Le Luong, Natalia Volodko, Orysya Svystun, Kayla Flood, Jonathan Lim, Miranda Sung, Jason R. B. Dyck, Chong Teik Tan, Yu-Chin Su, Victor C. Yu, John Mackey, Shairaz Baksh

**Affiliations:** From the Departments of ‡ Biochemistry and; § Pediatrics,; ¶ Cardiovascular Research Group, Faculty of Medicine and Dentistry, University of Alberta, Edmonton, Alberta T6G 2E1, Canada,; the ‖ Department of Pharmacy, Faculty of Science, National University of Singapore, Singapore 117543, Singapore,; the ** Department of Experimental Oncology, Cross Cancer Institute, University of Alberta, Edmonton, Alberta T6G 1Z2, Canada, and; the ‡‡ Cancer Research Institute of Northern Alberta, Edmonton, Alberta T6G 1Z2, Canada,; the §§ Alberta IBD Consortium, Edmonton, Alberta T6G 2X8, Canada, and; the ¶¶ Women and Children's Health Research Institute, Edmonton, Alberta T6G 1C9, Canada

**Keywords:** apoptosis, Bax, cancer, tubulin, tumor suppressor gene, ubiquitylation (ubiquitination), GWAS, Rassf1a, moap-1

## Abstract

Modulator of apoptosis 1 (MOAP-1) is a BH3-like protein that plays key roles in cell death or apoptosis. It is an integral partner to the tumor suppressor protein, Ras association domain family 1A (RASSF1A), and functions to activate the Bcl-2 family pro-apoptotic protein Bax. Although RASSF1A is now considered a *bona fide* tumor suppressor protein, the role of MOAP-1 as a tumor suppressor protein has yet to be determined. In this study, we present several lines of evidence from cancer databases, immunoblotting of cancer cells, proliferation, and xenograft assays as well as DNA microarray analysis to demonstrate the role of MOAP-1 as a tumor suppressor protein. Frequent loss of MOAP-1 expression, in at least some cancers, appears to be attributed to mRNA down-regulation and the rapid proteasomal degradation of MOAP-1 that could be reversed utilizing the proteasome inhibitor MG132. Overexpression of MOAP-1 in several cancer cell lines resulted in reduced tumorigenesis and up-regulation of genes involved in cancer regulatory pathways that include apoptosis (p53, Fas, and MST1), DNA damage control (poly(ADP)-ribose polymerase and ataxia telangiectasia mutated), those within the cell metabolism (IR-α, IR-β, and AMP-activated protein kinase), and a stabilizing effect on microtubules. The loss of RASSF1A (an upstream regulator of MOAP-1) is one of the earliest detectable epigenetically silenced tumor suppressor proteins in cancer, and we speculate that the additional loss of function of MOAP-1 may be a second hit to functionally compromise the RASSF1A/MOAP-1 death receptor-dependent pathway and drive tumorigenesis.

## Teaser

**Background:** MOAP-1 is a pro-apoptotic protein.

**Results:** MOAP-1 expression is reduced in cancers and high expression correlated with increased patient survival. MOAP-1 can associate with tubulin and modulate tubulin stability. Furthermore, loss of the BH3 domain of MOAP-1 resulted in lack of tumor suppressor function.

**Conclusion:** MOAP-1 is a highly regulated tumor suppressor protein.

**Significance:** The loss of MOAP-1 may significantly contribute to tumorigenesis.

## Introduction

MOAP-1 was first identified as a novel Bax-associating protein in a yeast two-hybrid protein screen (1, 2). It has been demonstrated to be involved in both the intrinsic and extrinsic pathways of cell death and is required for inducing Bax conformational change and mitochondrial localization to promote subsequent cell death (2, 3). MOAP-1 can also selectively associate with the anti-apoptotic proteins Bcl-2 and Bcl-X_L_ (1). We and others have demonstrated that MOAP-1 knockdown cells are resistant to a variety of apoptotic stimuli, including staurosporine, serum withdrawal, UV irradiation, tumor necrosis factor (TNF) α, and TNF-related apoptosis-inducing ligand (2–4). In nonapoptotic cells, MOAP-1 is an unstable protein (half-life ∼25 min) that is constantly turned over via the proteasome degradation pathway (5). However, during apoptotic stimulation, MOAP-1 expression is up-regulated in response to serum withdrawal, etoposide, TNF-related apoptosis-inducing ligand, or the endoplasmic reticulum stress inducer thapsigargin (5). MOAP-1 up-regulation under apoptotic stress can occur as a result of a tripartite motif containing 39 (TRIM39)-mediated inhibition of anaphase-promoting complex (APC/C^Cdh1^) E3 ubiquitin ligase-dependent polyubiquitination of MOAP-1 (5–7). Although it has been shown that MOAP-1 is degraded in a cell cycle-specific manner requiring the cell cycle regulator APC/C^Cdh1^ E3 ubiquitin complex, it remains unknown how this may be important for the control of apoptosis or cell proliferation (5, 6).

We and others have demonstrated association of the tumor suppressor, RASSF1A, with MOAP-1 and requirement of RASSF1A/MOAP-1 for TNF-R1-driven apoptosis (2, 4, 8). The loss of RASSF1A by small interfering RNA (siRNA) knockdown or genetic knock-out results in a significant loss of death receptor-dependent apoptosis with only minor effects on the intrinsic pathway of cell death (2). RASSF1 is involved in apoptosis, cell cycle control, regulation of microtubule stability, and tumor suppression and is epigenetically silenced in human cancers. RASSF1A can associate with MOAP-1 independent of the presence of K-Ras (2) in contrast to some reports (4). Similarly, RASSF6 has also been demonstrated to associate with MOAP-1, and the Hippo kinase, MST2, can interfere with this association to prevent RASSF6/MOAP-1-directed apoptosis. The importance of these observations physiologically is still unknown, but association between RASSF6 and MOAP-1 does not require the presence of activated K-Ras (9, 10).

In this study, we demonstrate that ectopic expression of MOAP-1 resulted in reduced formation of foci on soft agar and reduced proliferative capacity of H1299 cells. Furthermore, overexpressed MOAP-1 in several cancer cell lines resulted in reduced formation of tumors in a xenograft model in athymic nude mice suggesting a universal role as a tumor suppressor protein. Mechanistically, we can demonstrate that MOAP-1 may carry out its tumor suppressor function by the involvement in apoptosis (2, 8) and associations with tubulin isoforms to stabilize tubulin (this study). This will aid in sister chromatid separation in a similar manner to RASSF1A (11, 12). In addition, we provide evidence for involvement in DNA damage control and cell metabolism that may also influence control of tumor formation. These studies illustrate important links to RASSF1A (a *bona fide* tumor suppressor protein) and suggest that MOAP-1 synergizes with RASSF1A to inhibit tumorigenesis.

## Materials and Methods

### 

#### 

##### Antibodies and Reagents

Antibodies were purchased from the following vendors: p53 and Aurora B were from Abcam; mouse anti-ubiquitin (sc-8017), STAT3 (sc-482), STAT5a (sc-1081), GNB2L1/RACK1 (sc-17754), IRα (sc-710), β (sc-371), rabbit anti-γ-tubulin (sc-10732), mouse anti-α-tubulin (sc-8035) were from Santa Cruz Biotechnology; anti-RASSF1A (M304) was a gift from Dr. Gerd Pfiefer; PTEN (catalog no. 9552) and PARP^3^ (catalog no. 9542S), PKM2 (Y105, 3827S and total, catalog no. 4053S), GS3K-3β (catalog no. 9336S and total catalog no. 9315S), and AMPK (Thr-172, catalog no. 2531S; total, catalog no. 2532) were from Cell Signaling; MAP1S was kindly provided by Dr. Leyuan Liu (Texas A&M Health Science Center); mouse anti-β-tubulin was from Sigma (T-5201); mouse anti-acetylated α-tubulin was from Sigma (T-6793); ECL detection was from GE Healthcare (ECL RPN2106); and SYBR Green SuperMix was from Applied Biosystems (Foster City, CA).

##### Cell Culture and Transfection

Cells were grown and transfected with PEI as described previously (11, 13). Cells were lysed in SB lysis buffer (50 mm HEPES (pH 7.5), 150 mm NaCl, 1 mm MgCl_2_, 1.5 mm EDTA, 0.5% Triton X-100, 20 mm β-glycerol phosphate, 100 mm NaF, 0.1 mm PMSF) (11) or in standard RIPA buffer (8) as indicated. Apoptotic assays were carried as described previously (2). The cell lines utilized in this study included the following: HEMa-LP cells and melanoma cell lines from Dr. Sujata Persad (University of Alberta); breast cancer cell lines from Dr. Ing Swie Goping (University of Alberta); pediatric leukemia cell lines (Dr. Aru Narendran, University of Calgary); colon cancer cell lines (Dr. Eytan Wine, University of Alberta); ovarian cancer cell lines (Dr. YangXin Fu, University of Alberta), and neuroblastoma cell lines from Dr. Roseline Godbout (University of Alberta). HCT116 cells (containing endogenous RASSF1A and MOAP-1) were utilized for our xenograft assays as they transfect to >40%, maintain the expression of transiently transfected HA-RASSF1A for up to 10 days in culture, and produce tumors within 30 days (11). For most xenograft assays, the growth path is determined within the first 5–10 days. As such, even if expression is reduced, the growth will continue. If stables were not utilized, pools and not single clones expressing MOAP-1 were chosen.

##### Reverse Transcriptase (RT)-PCR

1 μg of RNA was treated with DNase I (Invitrogen) according to the manufacturer's instructions. RNA was then converted into cDNA with an Applied Biosystems high capacity cDNA reverse transcription kit according to the manufacturer's instructions. After reverse transcription, cDNA was diluted 10 times with RNase-free water, and 5 μl was used in PCRs using New England Biolabs *Taq*DNA polymerase with standard Taq buffer. PCR parameters were as follows: denaturation at 94 °C for 2 min (1 cycle), 94 °C for 1 min, 54 or 58 °C (for MOAP1 or GAPDH) for 1 min, and 68 °C for 1 min (35 cycles) followed by a final extension at 68 °C for 10 min. PCR products were analyzed on a 2% agarose gel and visualized with ethidium bromide. The followingprimers were used: MOAP1 forward 5′-ACATGAAAATGGCTCCTTAGAC-3′ and MOAP1 reverse 5′-GACACGAATAACATCAAGTGCT-3′; GAPDH forward 5′-CATGACAACTTTGGTATCGTG-3′ and GAPDH reverse 5′-GTGTCGCTGTTGAAGTCAGA-3′.

##### Genome-wide Association Study (GWAS) of Human Tumor Xenografts

HCT116 human tumor xenografts were established in athymic nude mice. At day 35, tumors were excised, stored in RNAlater solution (Qiagen), and used for RNA isolation according to the manufacturer's protocol (Qiagen RNeasy kits). RNA samples with RNA integrity numbers of >7.0 were used for gene expression analysis as described previously (14). Data normalization and analysis were performed using GeneSpring GX 11.5.1 (Agilent Technologies). Normalized data were log_2_ transformed and averaged over three independent tumor replicates per construct. The data discussed in this publication have been deposited in NCBI's Gene Expression Omnibus (55, 56) and are accessible through GEO Series accession number GSE43990 at www.ncbi.nlm.nih.gov.

##### Analysis of MOAP-1 Expression in Breast Cancer Database

Breast cancer microarray data were generated as described previously and provided to us through the courtesy of Dr. John Mackey (14). Significance of data was evaluated by Student's *t* tests (two-tailed), and receiver operating characteristic curve analysis was performed to establish the MOAP-1 expression cutoff.

##### Canonical Pathway and Biological Function Analysis of GWAS Expression Changes

Post-analysis of gene expression changes was performed using Ingenuity Pathway Analysis software (Ingenuity Systems). Functional analysis was performed to identify the biological functions most significant to the dysregulated molecules in our dataset with *p* values calculated by right-tailed Fisher's exact test. Canonical pathway analysis was employed to identify the pathways from the Ingenuity Pathway Analysis library that were most significant to our dataset.

##### Oncomine Meta-analysis of Human Cancer Microarrays

Differential expression analysis was performed for MOAP-1 in the normal *versus* cancer category using Oncomine cancer microarray database, version 4.4, Research Edition (Compendia Bioscience, Ann Arbor, MI). We assigned a cut threshold of fold-change ≥1.5 and *p* ≤ 0.05 for our meta-analysis and only included MOAP-1 expression data for studies that met this significance. Results were grouped based on cancer type and re-plotted as fold-changes in MOAP-1 mRNA levels relative to normal tissue.

##### Kaplan-Meier Survival Analysis

Kaplan-Meier survival curves for MOAP-1 and RASSF1A expression in 88 neuroblastoma patients were kindly generated for us by Dr. Rogier Versteeg (University of Amsterdam, Netherlands). mRNA levels of MOAP-1 and RASSF1A were quantified from 88 neuroblastoma patients, and Kaplan-Meier survival curves were generated to demonstrate the correlation between expression levels of MOAP-1 and RASSF1A with overall patient survival probability from neuroblastoma.

##### Statistical Analysis

All experiments were carried out at least three times. Significance of data was evaluated by performing a Student's *t* test (two-tailed) as indicated.

## Results

### 

#### 

##### Oncomine Database Reveals Reduced MOAP-1 Expression in Human Cancers

**FIGURE 1.**
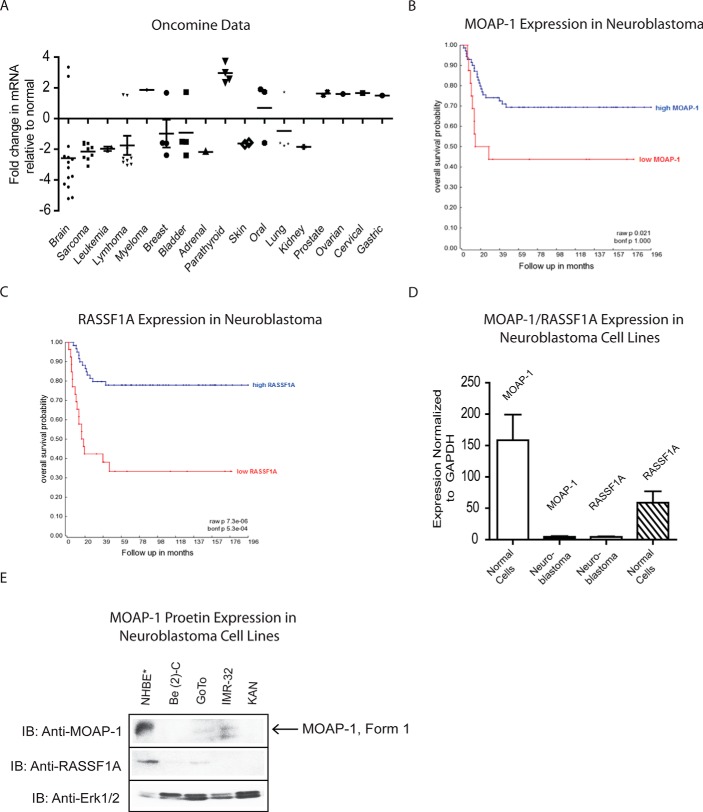
**Evidence of reduced MOAP-1 expression from cancer databases and in neuroblastoma.** *A,* differential expression analysis of MOAP-1 in normal *versus* cancer tissues from the microarray database Oncomine. Results from individual microarray studies are each represented by a single point on this plot (*n* = 48 within each study). The *y* axis depicts the fold-change in MOAP-1 mRNA levels relative to normal tissue for each of the multiple cancer types for which MOAP-1 expression data were available, as shown on the *x* axis. *Horizontal bars* represent the average MOAP-1 mRNA fold-change within that cancer type. All fold-changes are greater than or equal to 1.5, and *p* values are less than or equal to 0.05. *B* and *C,* Kaplan-Meier survival curves for neuroblastoma patients demonstrating a correlation between expression levels of MOAP-1 (*n* high expression = 72; *n* low expression = 16; expression cutoff, 700.1 for high expression *versus* low expression for MOAP-1 (arbitrary mRNA units indicated)) (*B*) and RASSF1A (*n* high expression = 61; *n* low expression = 27; expression cutoff, 101.7 (same comparison as for MOAP-1)) with overall patient survival probability from neuroblastoma (*C*). *D,* mRNA expression of MOAP-1 by qPCR 1 in normal (normal human bronchoepithelial, breast, and colon epithelial cells data were pooled) *versus* neuroblastoma cell lines (Be(2)c, GoTo, LAN-1, KAN, Nub7, SKNAS, and SH-SY5Y). *E,* protein expression of MOAP-1 in neuroblastoma cell lines as indicated. *IB,* immunoblot.

A meta-analysis of microarray data was performed using the on-line cancer microarray database Oncomine (Compendia Bioscience, Ann Arbor, MI) (15). Differential expression analysis of MOAP-1 was carried out in samples from normal *versus* malignant tissues with data subsequently compiled from microarray studies meeting the threshold fold-change ≥1.5 and *p* value ≤ 0.05. The results obtained from this meta-analysis indicate that MOAP-1 expression is markedly reduced in multiple types of human cancers (Fig. 1*A*), especially in the brain, breast, blood, skin, and lung suggesting that loss of MOAP-1 is important for tumorigenesis to occur. MOAP-1 may possess a potential tumor suppressor function in specific cell types. Interestingly, parathyroid, myeloma, prostate/ovarian/cervical (that is the reproductive organs), and gastric datasets revealed elevated expression of MOAP-1 to suggest that MOAP-1 expression can vary widely and may have differential function in numerous tissues. Please note that plots for “sarcoma” classification include the following: dedifferentiated liposarcoma, myxofibrosarcoma, round cell liposarcoma, and fibrosarcoma. The “leukemia” plots include B-cell childhood acute lymphoblastic leukemia, T-cell prolymphocytic leukemia (all underexpressed), and Hairy cell leukemia (overexpressed). The “lymphoma” plots include anaplastic large cell lymphoma (ALK-positive), primary cutaneous anaplastic large cell lymphoma, classical Hodgkin lymphoma, angioimmunoblastic T-cell lymphoma (all underexpressed), and mantle cell lymphoma (overexpressed).

##### Low MOAP-1 Expression in Neuroblastoma Patients Correlates with Decreased Survival Probability

Neuroblastoma is predominantly a pediatric form of cancer that arises from progenitor cells of the sympathetic nervous system and is the most common solid tumor in childhood (16). Remarkably, neuroblastomas display the highest rate of spontaneous regression among all human cancers possibly due to delayed activation of normal apoptotic signaling pathways (17). Because MOAP-1 is frequently down-regulated in brain cancer, it may therefore have a role in inhibiting malignancy formation in brain tissues. We explored MOAP-1 mRNA expression in 88 neuroblastoma patients in collaboration with Dr. Rogier Versteeg (University of Amsterdam, Netherlands). Kaplan-Meier survival analysis demonstrated that patients with high expression of either *MOAP-1* or *RASSF1A* experienced greater survival rates *versus* those with low expression of either gene (Fig. 1, *B* and *C*), thereby suggesting that both MOAP-1 and RASSF1A may behave as tumor suppressor proteins in the pathogenesis of neuroblastoma. We confirmed our database results by real time quantitative PCR (qPCR) in neuroblastoma cell lines (Fig. 1*D*) and by protein immunoblotting (Fig. 1*E*). Several neuroblastoma cell lines revealed 80–90% reduction of MOAP-1-Be(2)c (bone marrow disease derived from SK-N-Be(2)), GoTo (derived from stage IV disease), IMR-32 (derived from the abdominal mass metastatic site), SMS-KAN (KAN, derived from primary pelvic tumor) (Fig. 1*E*), Nub7 (MycN amplified), and SK-NAS (bone metastasis in origin) (data not shown) when compared with levels in normal human lung primary broncoepithelial (NHBE) cells. RASSF1A expression by qPCR was virtually absent due to epigenetic silencing (data not shown and see Refs. 12, 18). These data reinforce previous findings demonstrating frequent inactivation of RASSF1A and reduced expression of MOAP-1 to result in poor disease outcome (19, 20). Considering that both MOAP-1 and RASSF1A are essential components of death receptor-mediated apoptosis (2, 8), these observations suggest that the reduction of MOAP-1 and RASSF1A expression levels in neuroblastoma cells contributes significantly to poor patient prognosis and decreased survival.

##### Decreased MOAP-1 Expression in Breast Cancer Patients Correlates with Increased Cancer Aggressiveness

Currently, four major breast cancer subtypes can be distinguished based on the expressions of estrogen receptor (ER), progesterone receptor (PR), and Her2/Neu and are classified as the following in the order of increasing cancer aggressiveness: luminal A (ER+, PR+/−, and Her2−); luminal B (ER+, PR−/+, and Her2+); Her2-amplified (ER−, PR−, and Her2+); and triple negative or basal-like (ER−, PR−, and Her2−) (21). Luminal A breast cancers are the most common subtype that respond well to adjuvant hormone therapy and have the best overall prognosis. In contrast, there are currently no targeted therapies available for the treatment of triple-negative breast cancers that are associated with the worst overall and disease-free survival rates (21, 22).

**FIGURE 2.**
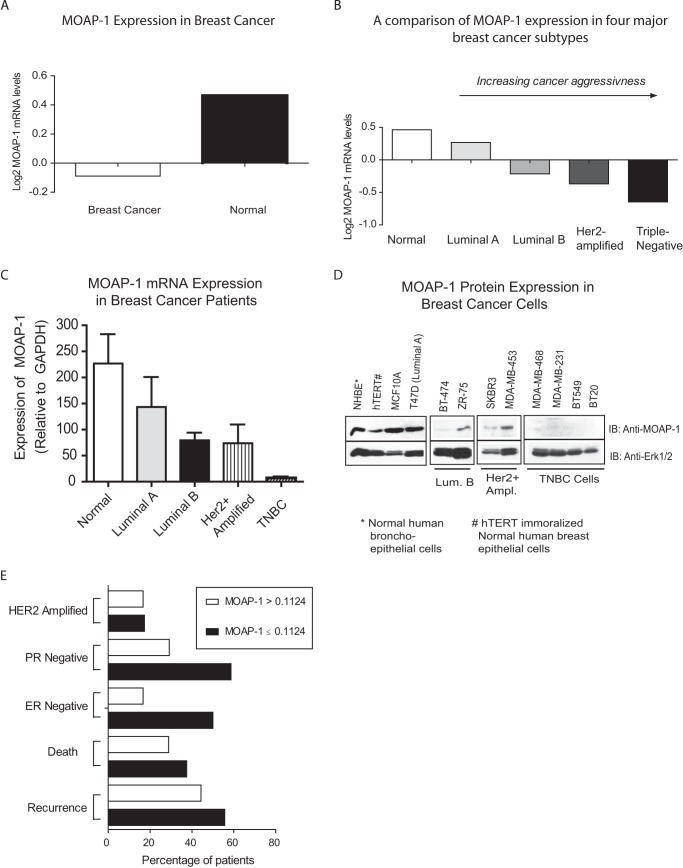
**Expression of MOAP-1 in breast cancer.** *A,* comparison of MOAP-1 mRNA levels in cancerous *versus* normal breast tissue (*n* = 10) and breast cancer (*n* = 176); *p* value = 2.15E-09. *B,* average MOAP-1 expression is significantly reduced in 3 out of 4 major breast cancer subtypes relative to normal breast tissue. (Luminal A: *p* value = 0.34, *n* = 75; Luminal B: *p* value = 0.0018, *n* = 19; HER2-amplified: *p* value = 0.0067, *n* = 8; Triple negative: *p* value <0.0001, *n* = 56; Normal: *n* = 10.) Note that 18 patients were excluded from this assessment because they could not be classified. “Normal” values were plotted from results using normal bronchial, breast, and colon epithelial cells. *C,* mRNA expression of MOAP-1 by qPCR in breast cancer patient subtypes as indicated (*n* = 6). *D,* protein expression of MOAP-1 in the indicated cell lines classified by breast cancer subtypes. *IB*, immunoblot. *E,* MOAP-1 expression is significantly reduced in ER-negative and PR-negative breast cancers. Breast cancer patient data were analyzed based on levels of MOAP-1 expression with relation to various clinical and pathological features of interest as shown. Receiver operating characteristic curve analysis calculated MOAP-1 expression cutoff at 0.1124 (low MOAP-1 expression ≤0.1124 and high MOAP-1 expression >0.1124). ER-negative breast cancers: *n* = 64, *p* value = 1.2E-06; PR-negative breast cancers: *n* = 82, *p* = 7.5E-05.

Breast cancer microarray expression data generated from 176 primary and treatment-naive breast cancer samples and 10 normal breast tissue samples (14) revealed that MOAP-1 expression was reduced 5-fold in breast tumor samples (Fig. 2*A*). Detailed analysis revealed a steady and significant decrease in MOAP-1 expression that correlated with increasing breast cancer aggressiveness in luminal B, Her2-amplified, and triple-negative subtypes (Fig. 2*B*). These changes were confirmed by MOAP-1 qPCR on patient tumor samples obtained from the Alberta Cancer Research Biobank (Fig. 2*C*) and from representative cell lines that reflect the categories in Fig. 2*B* (Fig. 2*D*). MOAP-1 expression was readily detected in the normal human lung primary broncoepithelial (NHBE) cell line, in the immortalized breast epithelial line hTERT (breast epithelial cells immortalized with human telomerase), and in the noninvasive cell line derived from a patient with fibrocystic disease of the breast, MCF-10A (Fig. 2*D*). In contrast, expression is reduced or not detectable in several breast cancer cell line subtypes as indicated in Fig. 2*D*.

All samples in Fig. 2, *C* and *D,* contain >40% of their RASSF1A promoter CpG island regions epigenetic silenced by methylation. Epigenetic silencing of RASSF1A in breast cancer is well documented and is one of the earliest detectable changes in breast cancer progression (12). These results suggest that MOAP-1 expression is down-regulated during breast cancer progression, may parallel the loss of RASSF1A, and subsequently contribute to the poor patient outcomes associated with more aggressive breast cancer subtypes. Although we did not observe an association between levels of MOAP-1 expression and breast cancer family history, recurrence, death, or Her2-amplification, we did discover a significantly higher proportion of patients expressing low MOAP-1 with ER-negative and PR-negative breast tumors (Fig. 2*E* (Her2+ amplified and TNBC) and Table 1). Hormone receptor-negative breast cancers carry a less favorable prognosis than tumors with hormone receptors and do not benefit from endocrine therapies (23). Therefore, the loss of the pro-apoptotic MOAP-1 protein may contribute significantly to the less favorable prognosis than tumors with hormone receptors.

**TABLE 1:** **MOAP-1 expression is significantly reduced in ER-negative and PR-negative breast cancers** Breast cancer patient data were analyzed based on levels of MOAP-1 expression with relation to various clinical and pathological features of interest as shown. Region of choice curve analysis was calculated for MOAP-1 expression cutoff at 0.1124 (low MOAP-1 expression [ltequ]0.1124 and high MOAP-1 expression >0.1124). ER-negative breast cancers: *n* = 64, *p* value = 1.2E-06; PR-negative breast cancers: *n* = 82, *p* value = 7.5E-05.

	No. of patients with low MOAP-1 (log2 mRNA ≤0.1124)	No. of patients with high MOAP-1 (log2 mRNA >0.1124)	*p* value
Total no. of patients	104	72	
Cancer recurrence	49	39	0.36071
Death	30	27	0.23622
Negative ER status	52	12	1.2E-06
Negative PR status	61	21	7.5E-05
HER2 amplified	18	12	0.9119
Breast cancer family history	45	32	0.85813

##### Reduced Expression of MOAP-1 in Cultured Cancer Cells

**FIGURE 3.**
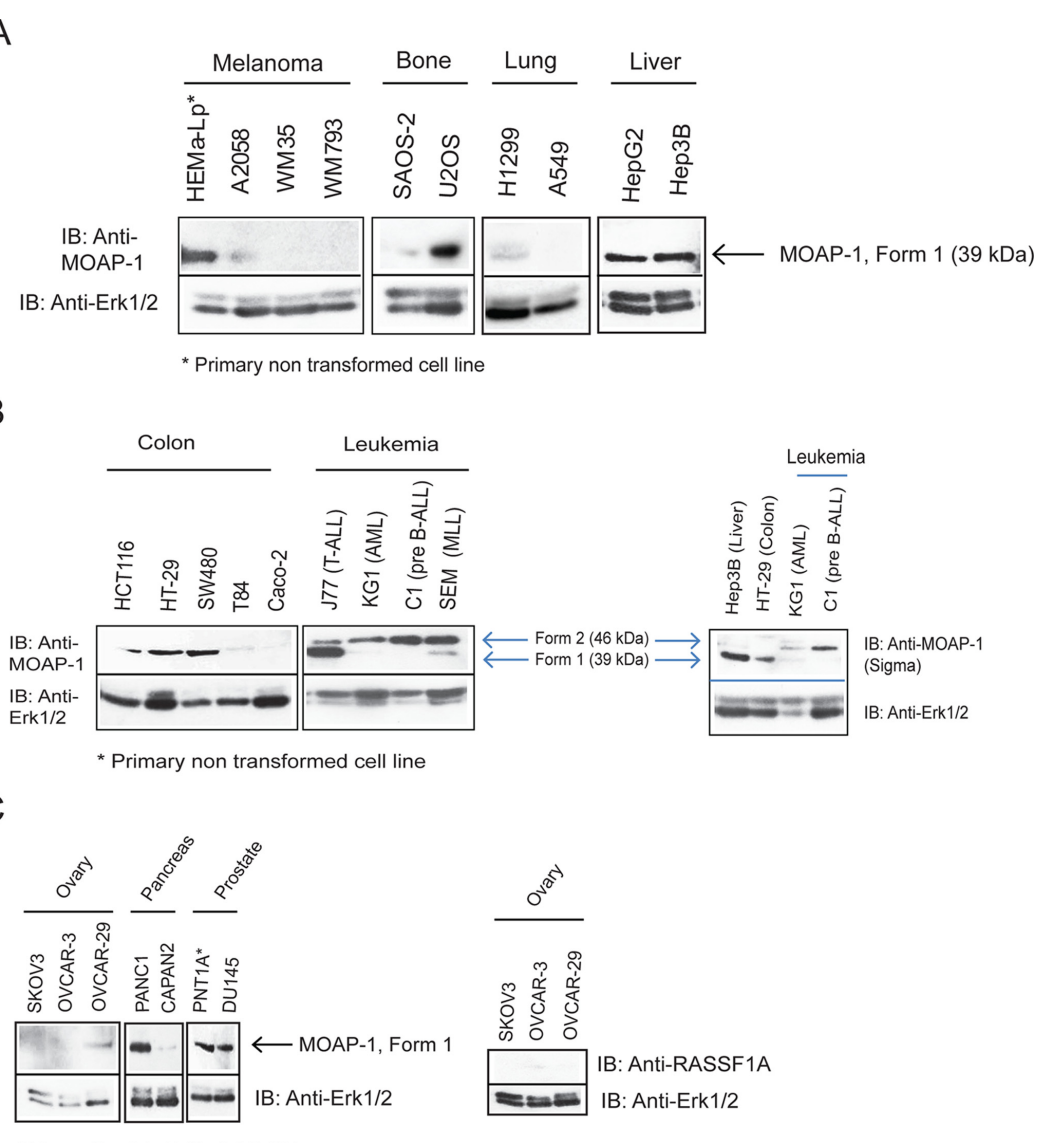
**Protein expression of MOAP-1 in the indicated cancer cell lines (*A–C*).** ERK1/2 is shown as a loading control for the lanes, and all lanes marked withan *asterisk* are normal cells. *IB,* immunoblot. Two forms of MOAP-1 can be observed blood cancers that migrate at ∼46 kDa instead of the normal 39 kDa. This result has been confirmed using the rabbit anti-MOAP-1 (from QED Biosciences) and rabbit anti-MOAP-1 from Sigma as indicated (*B, right side*).

In addition to detectable expression of MOAP-1 in NHBE, hTERT (immortalized with human telomerase), and MCF-10A, we can also detect MOAP-1 in other nontransformed cell lines such as HEMA-LP and PNT1A (Fig. 3, *A–C*). MOAP-1 protein was reduced or absent in many cancer cells as shown in Figs. 2*D* and 3. Noticeably, MOAP-1 expression was detected in the p53-positive cell lines (U2OS, HCT116, ZR-75, IMR-32, and PANC1) but was reduced/absent in p53-null cells (SAOS-2 and SKOV3), p53 frameshift mutation or rearrangement cell lines (Caco-2 and CAPAN-2), and p53-mutant cell lines (SKBR3, MDA-MB-468, MDA MB-231,BT-549, and BT-20 (breast cancer cells) (Fig. 2*D*), and OVCAR-3 (ovarian cancer). However, the expression of MOAP-1 did not always correlate with the status of p53 as SW480 (p53-null) has detectable MOAP-1, whereas the p53 wild type cell lines, A549, and the majority of the neuroblastoma cell lines do not have robust detection of MOAP-1. Among the cell lines that are *p53*^+/+^*MOAP-1*^−/−^, all have epigenetic silencing of RASSF1A (12). Therefore, we suspect that RASSF1A may influence the expression of MOAP-1, and we are currently exploring this unexpected observation. The dual loss of MOAP-1 and RASSF1A expression may contribute to malignant cell growth in the solid cancers.

In contrast to the solid cancers, MOAP-1 in several blood cancer cell lines revealed two distinct forms as detected by two independent antibodies (Fig. 3*B*, *right side*). The slower migrating 46-kDa form (form 2) of MOAP-1 can be found in cancers originating from the blood as ALL or AML subtypes (Fig. 3*B*).^4^ We are currently investigating why MOAP-1 appears as a slower migrating band in blood cancers, but we suspect that it may involve phosphorylation of MOAP-1 by an unidentified kinase.

##### Differential MOAP-1 Expression in Cancer Cells May AlsoBe Controlled by Post-translational Ubiquitination

**FIGURE 4.**
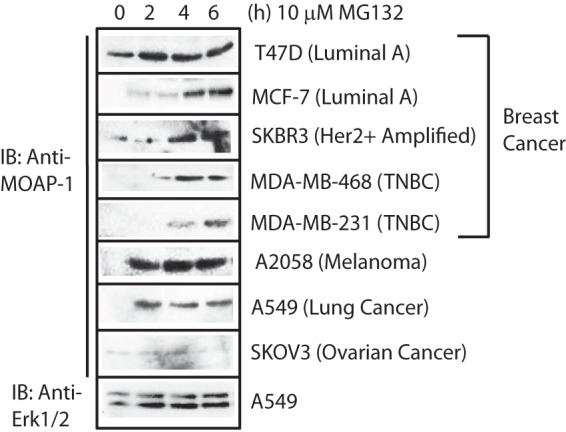
**MOAP-1 is rapidly turned over in several human cancer cell lines.** Immunoblotting (*IB*) for MOAP-1 was performed in selected cell lines by the addition of the proteosome inhibitor, MG132, at a concentration of 10 μm for the indicated times.

Exon sequencing revealed no mutations in MOAP-1 in several cancer lines investigated, including HCT116 colon cancer cells, H1299 small cell lung cancer, and MDA-MB-231 breast cancer cells. Quantitative PCR revealed decreased mRNA production for MOAP-1 in several cancer cells and patient tumor tissues (Figs. 1*D* and 2*C*). It is known that MOAP-1 appears to be highly turned over every 25 min via the ubiquitin-mediated proteasomal degradation (5) and thus may explain lack of detection. If MOAP-1 is regulated by ubiquitin-directed degradation, then the proteasome inhibitor, MG-132, should inhibit the degradation of MOAP-1 and stabilize its expression. Indeed, we can observe this with MG-132 treatment of several cell lines that initially did not reveal detectable MOAP-1 expression, including MDA-MB-468, MDA-MB-231, MCF-7, A549, and A2058 (Fig. 4). For the most part, MOAP-1 ubiquitination can be detected in most of the cell lines in Fig. 4 (data not shown) and are in line with the observations of Lee *et al.* (5). This would suggest that ubiquitin-directed modulation of MOAP-1 expression can occur in several cancers. Curiously, under MG-132 treatment, MOAP-1 expression in SKOV3 cells is barely detectable for unknown reasons. These experiments reveal the complexity of MOAP-1 regulation. We are currently investigating the importance of ubiquitination for MOAP-1 biology, the mechanism by which this occurs, and characterizing the importance of two identified E3 ligase interacting proteins with MOAP-1 and how they may control the biology of MOAP-1.

##### MOAP-1 Inhibits Cell Proliferation in Culture and Promotes Cell Death in Cancer Cells

**FIGURE 5.**
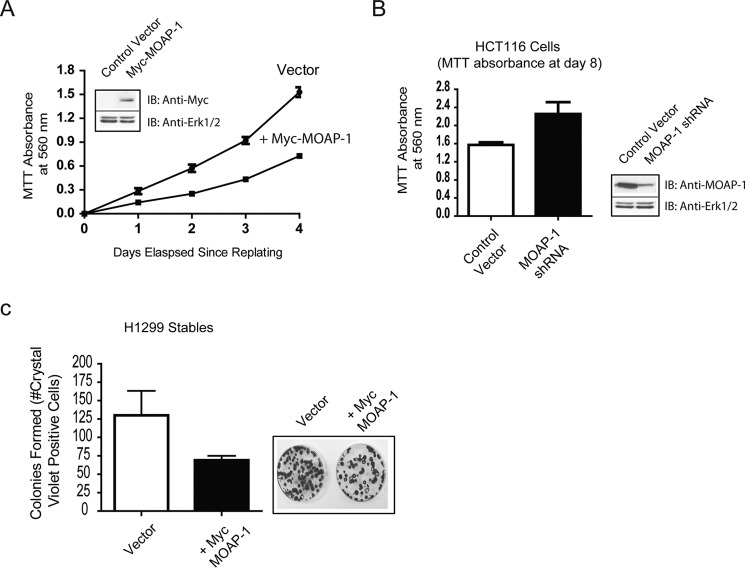
**MOAP-1 can inhibit cell proliferation *in vitro*.** *A* and *B,* MOAP-1 suppresses H1299 cell growth in culture. H1299 cells stably expressing vector or Myc-MOAP-1 (*A*) or MOAP-1 shRNA (*B*) were seeded at 4000 cells per well in a 96-well plate, and an MTT assay was carried out as outlined under “Experimental Procedures.” *Inset* represents immunoblot to confirm the expression of Myc-MOAP-1 with ERK1/2 as a loading control. *A, p* value = 0.0006, *n* = 16; *B, p* value = 0.0022, *n* = 8. *B*, *right side,* immunoblot to confirm shRNA knockdown of endogenous MOAP-1. *IB*, immunoblot. *C,* colony formation was carried out in H1299 stable cells expressing the indicated constructs. *p* value = 0.0361 and *n* = 4 −5 for both. H1299 cells were seeded at 600 cells per plate and allowed to grow for 11 days prior to staining with crystal violet. Number of cell colonies were counted by visual examination. The expression of Myc-MOAP-1 can be detected in these cells for >30 days after stables are formed (see Fig. 7*C* for stable expression of MOAP-1 in H1299).

A fundamental property that is shared by many tumor suppressor proteins is the ability to negatively regulate cell growth and proliferation. H1299 non-small cell lung carcinoma cells stably expressing Myc-MOAP-1 and HCT116 colon cancer cells transiently expressing shRNA to MOAP-1 were generated. Stable expression in H1299 cells was obtained using G418-resistant pools of cells expressing MOAP-1 to avoid effects of clonal variation. Using the MTT colorimetric assay for cell proliferation (24), we observed that cells expressing Myc-MOAP-1 were capable of inhibiting cell proliferation, whereas cells with reduced MOAP-1 expression promoted cell proliferation (Fig. 5, *A* and *B*). Colony formation assay also revealed the growth-suppressive function of MOAP-1 (Fig. 5*C*). This would suggest importance in inhibiting tumor formation.

**FIGURE 6.**
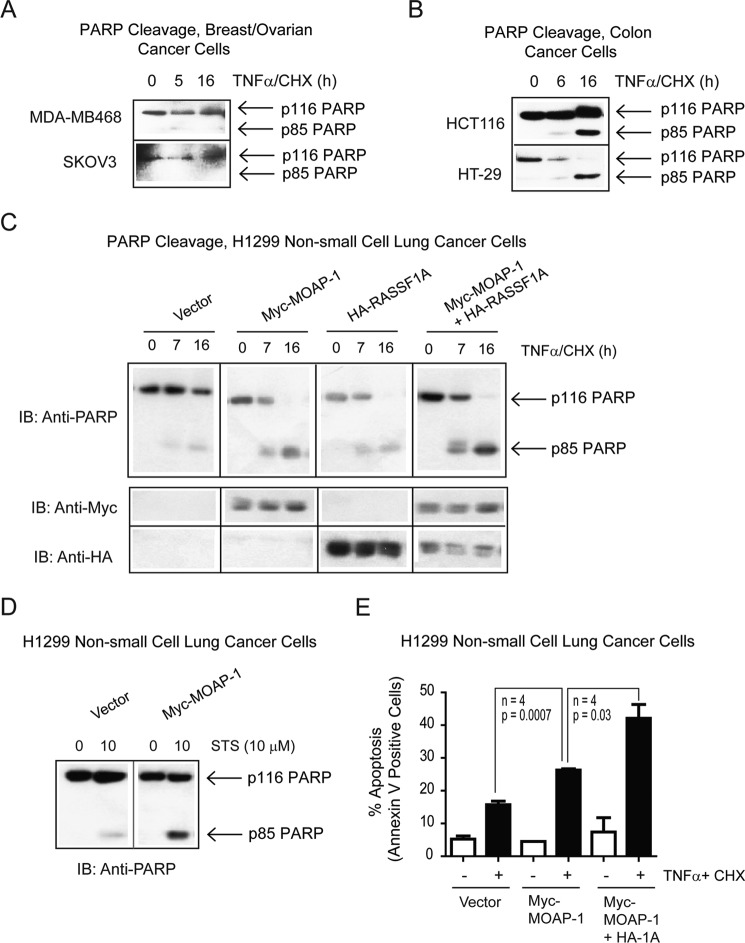
**Defective cell death in the absence of endogenous MOAP-1.** *A–C,* PARP cleavage was analyzed in response to TNFα/CHX in the indicated cancer cells. *IB,* immunoblot. *D*, in addition, cell death was also analyzed by staurosporine treatment of H1299 cells. Stable pools of Myc-MOAP-1-expressing H1299 cells were established for *D* and *E. E,* annexin-V staining in H1299 stable cells with the indicated expression constructs. All proteins were expressed at similar levels (data now shown). *n* = 3, *p* values as indicated.

Further analysis of several MOAP-1-negative and -positive cells verified the important role of MOAP-1 in growth suppression by promoting apoptosis (Fig. 6). Cells having robust expression of MOAP-1 (HCT116 and HT-29 colon cancer cells) had robust cleavage of poly(ADP-ribose) polymerase (PARP) upon TNFα/CHX (extrinsic pathway) stimulation, whereas cells with reduced or absent endogenous MOAP-1 expression (MDA-MB-468, SKOV3 and H1299 cells) displayed very limited PARP cleavage upon TNFα/CHX stimulation (Fig. 6, *A* and *B*). Upon stable re-expression of MOAP-1 in H1299 cells, PARP cleavage was significantly enhanced, and the p85-cleaved form of PARP was detected upon TNFα/CHX addition that appeared to be augmented by the presence of stably expressed RASSF1A and MOAP-1 (Fig. 6*C*). Furthermore, staurosporine (intrinsic apoptotic pathway stimulation) was also more robust in the presence of stably expressed MOAP-1 in H1299 cells (Fig. 6*D*). Annexin V staining confirmed our PARP results to illustrate that both early (annexin V staining) and late (PARP cleavage) markers of apoptosis were generated in the presence of MOAP-1 (Fig. 6*E*). Similar results were obtained for A549 lung cancer cells and SKOV3 ovarian cancer cells (data not shown). Together, these results demonstrate tumor suppressor properties of MOAP1 via repressing cell proliferation and promoting cell death in cancer cell lines.

##### MOAP-1 Inhibits Tumor Formation in Vivo via Its Pro-apoptotic Function

**FIGURE 7.**
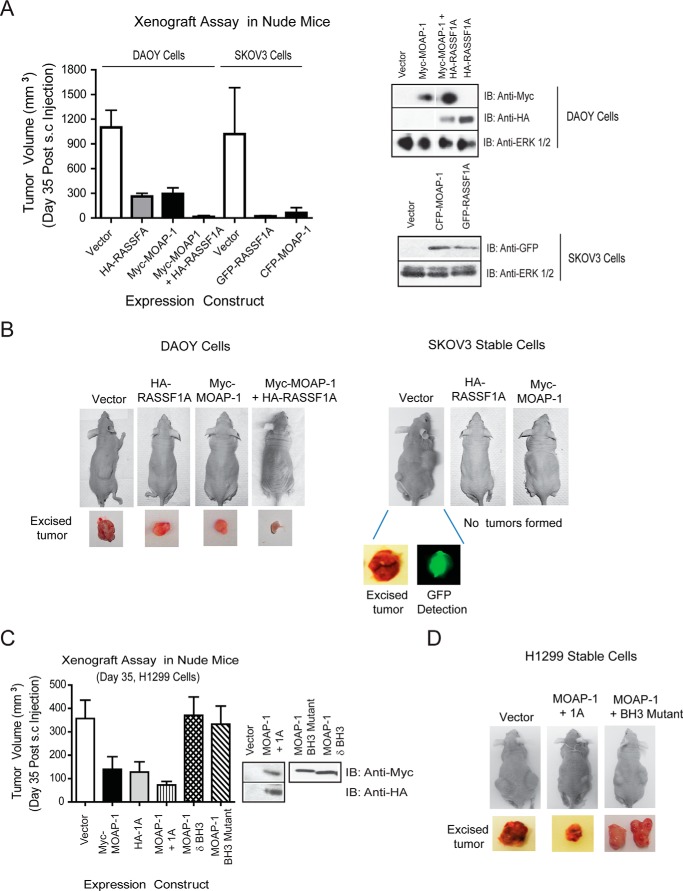
**MOAP-1 can inhibit tumor formation *in vivo*.** Xenograft assays were carried out in DAOY medulloblastoma and SKVO3 ovarian cancer cells (*A* and *B*) and in H1299 lung cancer cells (*C* and *D*). Stable cells were utilized for H1299 and SKOV3 cells, although DAOY cells contained transiently transfected expression vectors. For SKOV3, the anti-GFP antibody recognized both CFP-MOAP-1 and GFP-1A. For all, cells were harvested 48 h post-transient transfection, resuspended in Matrigel, and subcutaneously injected into male athymic mice. Immunoblots (*IB*) beside the graphs represent stable or transient expression of the indicated proteins. Athymic nude mice (Taconic Laboratories number NCRNU-M, CrTac:NCr-FoxN1Nu) were utilized for this assay as described previously (11). *A*, for DAOY cells, the *p* value between vector and HA-RASSF1A is 0.0003; the *p* value between vector and Myc-MOAP-1 or vector and Myc-MOAP-1 plus HA-RASSF1A is 0.0023 and 0.0065 respectively; *n* = 6–10 for all. *A*, *right side*, expressions of the indicated constructs are shown. For SKOV3, the *p* value between vector and HA-RASSF1A and vector and Myc-MOAP-1 was 0.009 and 0.004, respectively; *n* = 6 for all. *C*, *p* value between tumors formed in vector *versus* MOAP-1 cells was 0.0075, vector and RASSF1A was 0.0214, and vector and RASSF1A/MOAP-1 was 0.0049 (*n* = 8–10 for all). *p* value between MOAP-1 wild type and δBH3 or BH3 mutant is <0.027 and <0.0327, respectively.

**FIGURE 8.**
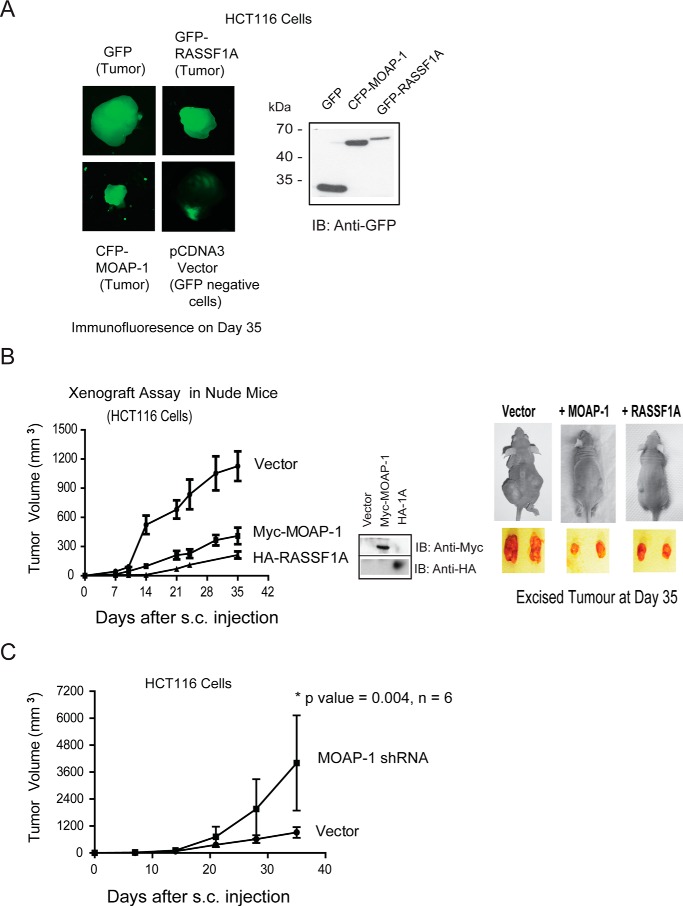
**MOAP-1 can inhibit tumor formation driven by HCT116 colon cancer cells *in vivo*.** HCT116 colon cancer cells with overexpressed proteins (*A* and *B*) or with shRNA to MOAP-1 (*C*) were generated by transient transfection with the indicated expression vectors. Cells were prepared and injected into nude mice as in Fig. 9. For HCT116 cells, the *p* value between vector and HA-RASSF1A and vector and Myc-MOAP-1 was 0.0026 and 0.0043, respectively; *n* = 10–16 for all. *B*, *middle*, expression of the indicated constructs are shown as well as mouse and tumor images, *B*, *far right,* representative tumors at day 35 of tumor growth. *A*, tumor images were captured using a multispectral FX instrument from Carestream to support the use of stable expression of GFP constructs for this assay. MOAP-1 shRNA in *C* results in >70% loss of MOAP-1 expression as presented.

To directly validate the tumor suppressor function of MOAP-1 *in vivo*, xenograft tumor assays were performed in athymic nude mice lacking a functioning immune system (25). To carry out these assays, we selected cancer cell lines with reduced or absent MOAP-1 expression (DAOY medulloblastoma cells, SKOV3 and H1299) or with a detectable amount of MOAP-1 (HCT116 cells). Myc-MOAP-1 was transiently expressed in DAOY medulloblastoma cells, SKOV3 (stable expression, ovarian cancer) (Fig. 7, *A* and *B*), or H1299 (stable expression, lung cancer, Fig. 7, *C* and *D*) and transiently in HCT116 colon cancer cells (Fig. 8, *A* and *B*). Following subcutaneous injection of these cells into the left and right flanks of athymic mice, a significant difference in the growth of tumors containing overexpressed Myc-MOAP-1 emerged for all cell lines tested when compared with tumors containing a control vector. This suggested a role for MOAP-1 in growth suppression. Interestingly, the loss of the function of the BH3 domain of MOAP-1 (either as a deletion or mutation) resulted in the inability to suppress tumor formation to suggest a significant dependence of MOAP-1 on its pro-apoptotic function to carry out tumor suppression (Fig. 7, *C* and *D*). Complementary to our overexpressed cells, shRNA knockdown of MOAP-1 resulted in a significant increase in tumor formation following subcutaneous injection of HCT116 cells containing shRNA to MOAP-1 (Fig. 8*C*). Not surprisingly, tumor formation was additionally reduced in the presence of HA-RASSF1A (for DAOY and H1299 cells, Fig. 7, *B* and *C*) suggesting a requirement for the RASSF1A/MOAP-1 apoptotic pathway in inhibiting tumor formation in these cell lines.

##### MOAP-1 Can Associate with Tubulin to Possibly Inhibit Tumor Formation

**FIGURE 9.**
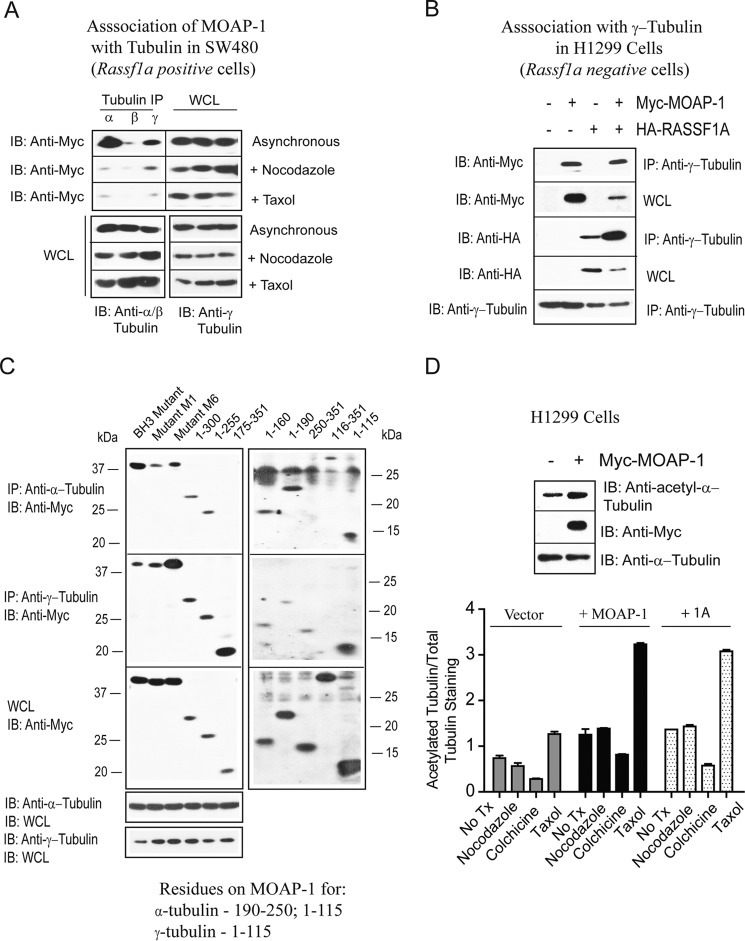
**MOAP-1 can associate robustly with tubulin isoforms, may not require RASSF1A for this association, and can stabilize microtubules.** Association of MOAP-1 with tubulin isoforms in SW40 cells (*A*), in H1299 cells (*B*), and in SW480 cells (*C*) used previously published mutants of MOAP-1 as indicated. *D,* acetylation status of α-tubulin−/+ Myc-MOAP-1 was determined with an anti-acetyl-α-tubulin antibody as indicated and quantified in the *bottom panel. Bottom panel*, total lysates of H1299 cells −/+ MOAP-1 or 1A were immunoblotted (*IB*) for acetylated tubulin followed by reprobing with an α-tubulin antibody to total tubulin content. Densitometry was carried on the immunoblots using the ImageJ software and plotted as shown. Expression of Myc-MOAP-1 and HA-1A was comparable (data not shown), and *n* = 3 for each point was independently carried out. *IP*, immunoprecipitation; *WCL*, whole cell lysate; *Tx*, treatment.

RASSF1A can physically associate with microtubules and with α-, β-, and γ-tubulin (11, 26), and the lack of microtubule association promotes tumor formation (11, 27). It has been demonstrated to co-localize with α-, β-, and γ-tubulin and influence the stability of α-tubulin in several cell types in the *Rassf1a*^−/−^ knock-out mouse embryo fibroblasts (11, 26, 28). RASSF1A was also found to stabilize γ-tubulin at the metaphase plate and serve as an important component of sister chromatid separation (28, 29) and the appearance of aneuploidy. Thus, association with tubulin is an important aspect of the tumor suppressor role for RASSF1A and possibly MOAP-1 due to the intimate connection between these two pro-apoptotic proteins. We thus explored whether MOAP-1 can associate with α-, β-, and γ-tubulin in the absence and presence of RASSF1A. SW480 and H1299 cells were utilized as they are RASSF1A-positive and -negative cell lines, respectively. Association of MOAP-1 with α- and γ-tubulin was readily detected in asynchronous cells as well as nocodazole- and taxol-treated cells (albeit at a much lower stoichiometry, Fig. 9*A*). In comparison, RASSF1A can associate equally with tubulin isoforms (11). Furthermore, the association with γ-tubulin was independent of the presence of RASSF1A (Fig. 9*B*). Using deletion constructs to MOAP-1, we were able to identify residues 1–115 as important for γ-tubulin association, whereas residues important for α-tubulin association were indeterminate. MOAP-1 expression constructs 1–115, 1–160, and 1–190 can associate with α-tubulin, although constructs 250–351 cannot (Fig. 9*C*). It may well be that α-tubulin docks to multiple regions on MOAP-1. Interestingly, the MOAP-1 mutants in the BH3 domain, the M1 mutant (that lacks RASSF1A association (2)), or the mutant M6 (that lacks TNF-R1 association) do not interfere with either α-tubulin or γ-tubulin associations (Fig. 9*C*). The association of MOAP-1 with α-tubulin resulted in greater stability of α-tubulin in asynchronous H1299 cells that have a low detectable level of MOAP-1 (Fig. 9*D*). Furthermore, the presence of overexpressed MOAP-1 or RASSF1A resulted in greater stabilization of tubulin by taxol, resistance to nocodazole, or colchicine destabilization of tubulin (as monitored by its acetylation status, Fig. 9*D*, *bottom graph*). Thus, similar to the ability of RASSF1A to promote the stability of tubulin, MOAP-1 can also achieve this. This would suggest the importance of both RASSF1A and MOAP-1 to tubulin stability, organization of spindle formation at the metaphase plate, and the effective separation of sister chromatids during mitosis to reduce the incidence of aneuploidy and fulfill its function as a tumor suppressor protein.

##### Transcriptome Analysis of MOAP-1-overexpressing Tumors Reveals Novel Involvement in Several Molecular Pathways

**FIGURE 10.**
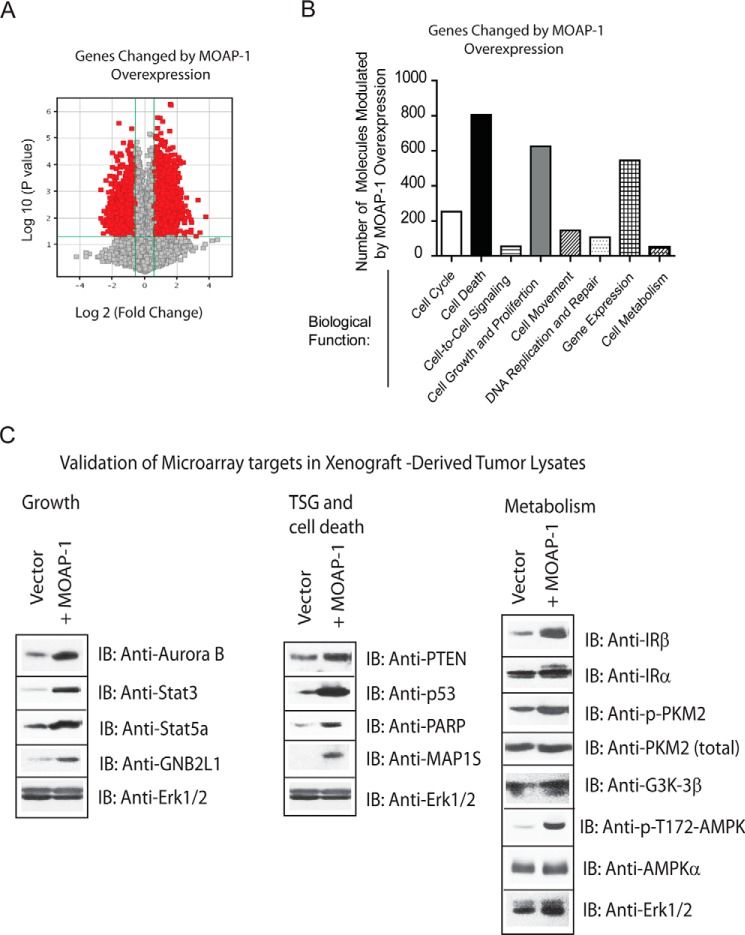
**Genes differentially modulated by the transient overexpression of Myc-MOAP-1.** *A, volcano* plot of the distribution of genes differentially regulated by Myc-MOAP-1 overexpression. *B, histogram* plot of the total number of gene expression changes associated with the indicated biological function in MOAP-1-overexpressing HCT116 tumor cells. *C,* validation of a few selected genes from Table 3. *IB*, immunoblot.

The role for MOAP-1 in cell death is well documented. MOAP-1 is known to partner with TNF-R1, RASSF1A, and Bax to promote apoptosis (1, 2) and partner with TRIM39 for protein stabilization (5). Although cell death and microtubule stability are important aspects of MOAP-1 biology, other possible mechanisms may exist to explain how MOAP-1 inhibits cell proliferation and tumor formation. To gain insight into these other mechanisms of tumor suppression, gene expression profiling was performed on MOAP-1-overexpressing xenograft tumors in nude mice. All experiments were carried out by transient transfections in HCT116 cells as described previously (11). Using this method, we were able to obtain >60% expression by day 2 that is sustained until day 10 after transfection in tissue culture (11). Because most of the tumor growth initiates between 7 and 14 days (as seen in Fig. 8, *B* and *C*), we postulate that >60% of the HCT116 cells expressing Myc-MOAP-1 will be sufficient to direct a growth path toward tumor suppression. RNA was extracted from resulting tumors and subjected to GWAS using the Agilent platform. GWAS revealed a total of 1434 differentially expressed genes by overexpression of MOAP-1 or, most likely, as a consequence of the biological ability of MOAP-1 to induce cell death and other physiological processes (Fig. 10*A*). Using the “core analysis” function in Ingenuity Pathway Analysis (Ingenuity Systems) (30), we were able to interpret gene expression changes in the context of biological functions and signaling pathways. Table 2 and Fig. 10*B* display several biological functions and signaling pathways identified as being most significant to the dataset of vector *versus* wild type MOAP-1 based on Fisher's exact test. Table 3 represents a selection of differentially expressed genes whose expression is modulated as a consequence of MOAP-1 overexpression and enhanced pro-apoptotic function, some of which encode potential growth-regulatory or tumor-suppressor functions. Cell death (805 molecules), cell growth and proliferation (625 molecules), and gene expression (545 molecules) were among the top biological functions associated with the dysregulated molecules (Fig. 10*B*).

**TABLE 2:**
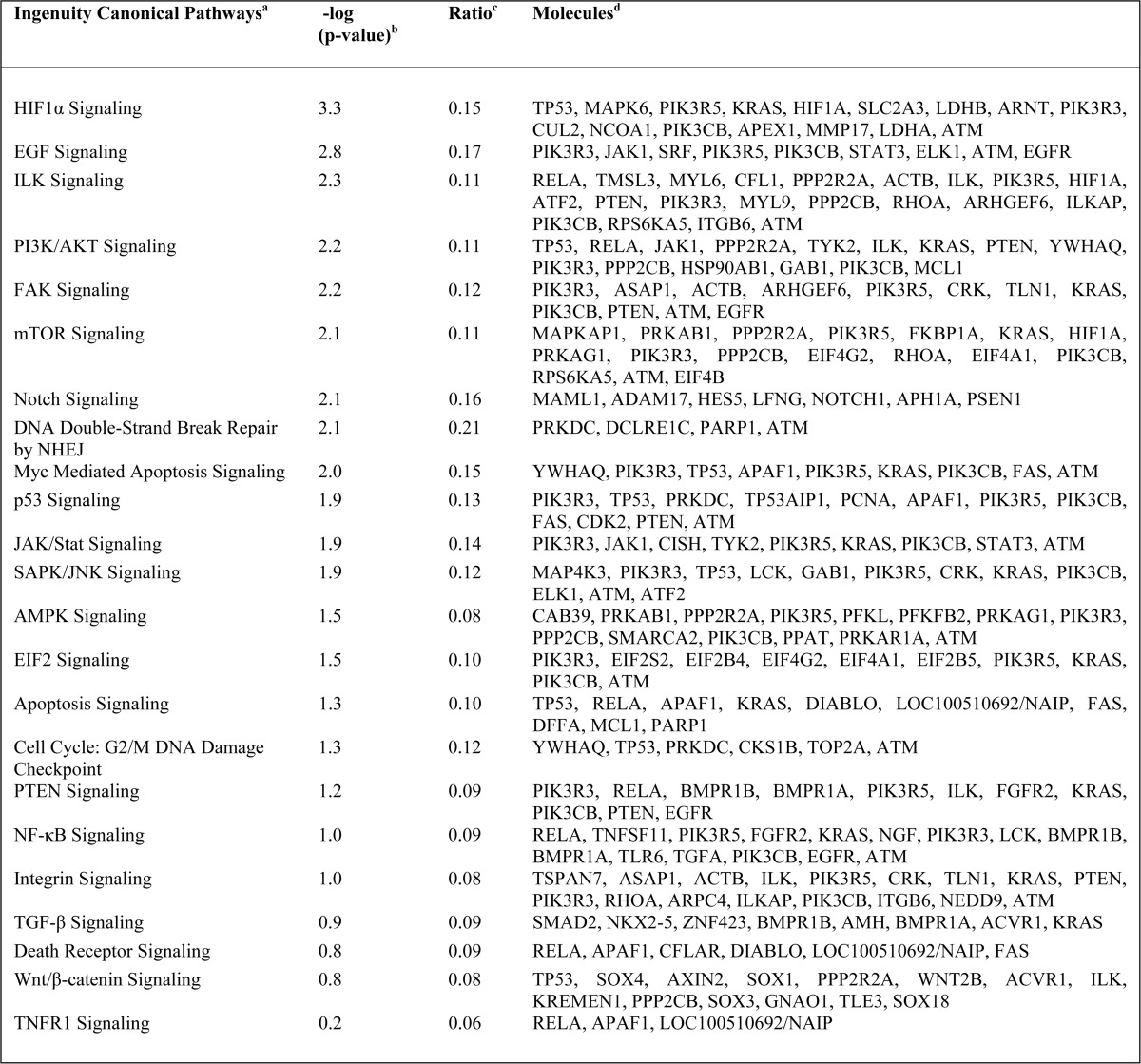
**Potential canonical pathways dysregulated by overexpressed Myc-MOAP-1 (relative to vector)**

*^a^* Ingenuity canonical signaling pathways that were most significant to the WT MOAP-1 *versus* vector control HCT 116 xenografts dataset were assigned from the Ingenuity Pathway Analysis library of canonical pathways.

*^b^* −log(p-value) ≥1.3 is equivalent to p-value ≤0.05.

*^c^* Ratio gives the number of dataset molecules that meet cut criteria in a given pathway divided by the total number of known molecules in that pathway.

*^d^* Molecules from the dataset that meet cut criteria and are involved in the corresponding signaling pathway.

**TABLE 3:**
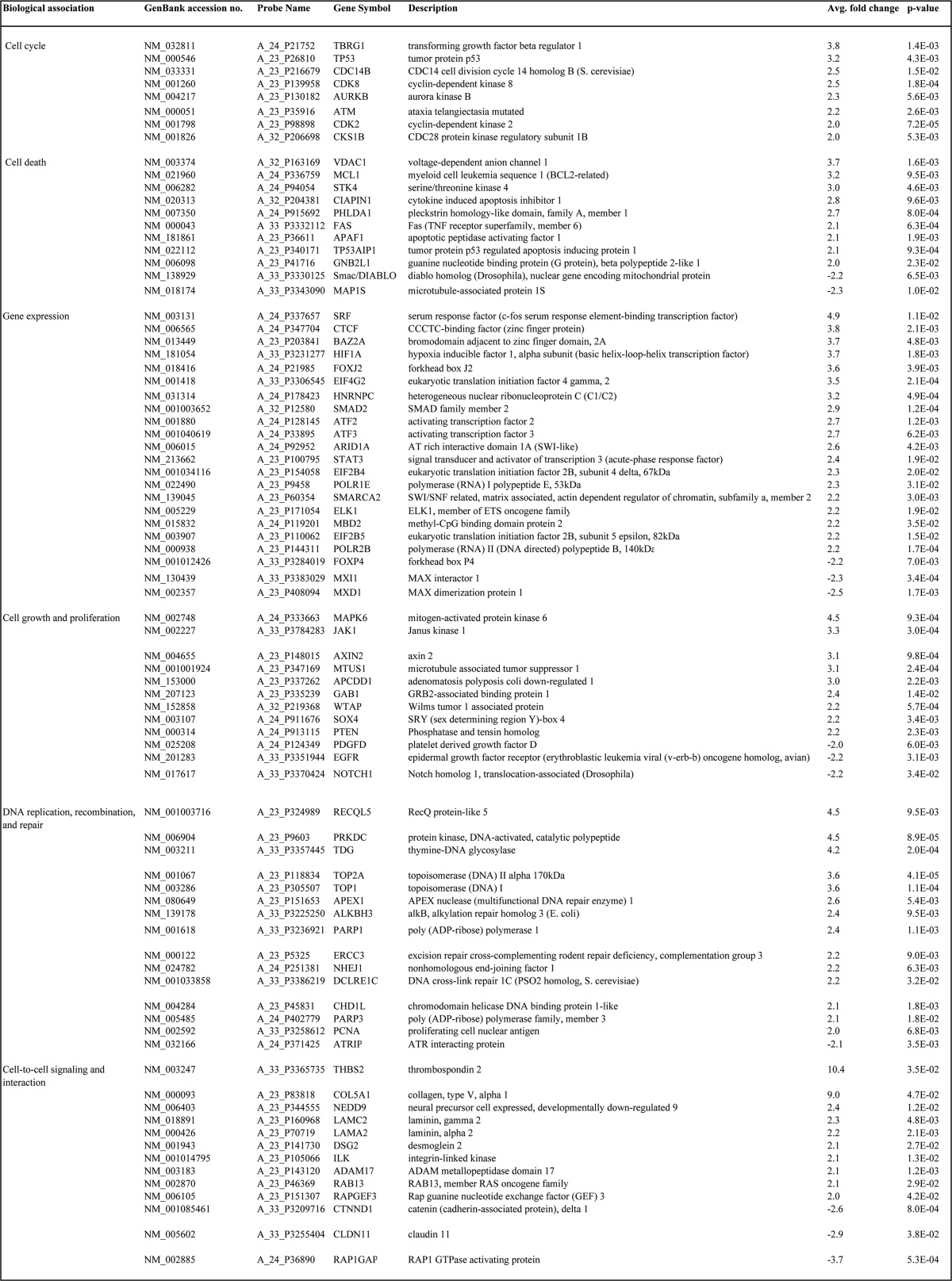
**Selected genes differentially expressed by overexpressed Myc-MOAP-1 (relative to vector control)**

Of particular interest, *TP53,* encoding the tumor suppressor protein p53, was found to be up-regulated by over 3-fold in the presence of wild type MOAP-1 relative to vector (Table 3 and validated in Fig. 10*C*). Several molecules involved in p53-dependent signaling pathways were also present at elevated levels (Table 3). These include cyclin-dependent kinase 8 (CDK8) (31), cell division cycle 14 homolog B (CDC14B) (32), and transforming growth factor β regulator 1 (TBRG1/NIAM) (33). In addition, p53 transcriptional targets such as proliferating cell nuclear antigen (34), Fas (35), and epidermal growth factor receptor (36) were also found to be differentially expressed (Table 3). Together, these gene expression changes may be involved in the maintenance of genomic integrity and protection against uncontrolled cell proliferation. In addition, other molecules from p53-dependent signaling pathways (not directly related to proliferation and cell death) were also found to be elevated with wild type MOAP-1 overexpression. These include hypoxia-inducible factor 1α subunit (HIF1α) and the G_2_/M DNA damage checkpoint kinase ataxia telangiectasia mutated (ATM, see Table 3). Whether or not MOAP-1 may be involved in the cellular responses to hypoxia or DNA damage warrants further investigation.

The overexpression of MOAP-1 also resulted in the up-regulation of several pro-apoptotic kinases connected to RASSF1A, including mammalian ste20-like kinase 1 (MST1) (mammalian *Hippo*) and aurora kinase B (Table 3 and validated in Fig. 10*C*). The activity of MST1 is initiated by association with RASSF1A (37), and aurora kinase B can directly phosphorylate RASSF1A to promote its degradation during mitosis via the anaphase-promoting complex/cyclosome (APC/C) (38, 39). Currently, the influence of MOAP-1 in the Hippo pathway regulation or APC/C function remains unknown, but our GWAS study would suggest this and thus warrants further investigation.

**FIGURE 11.**
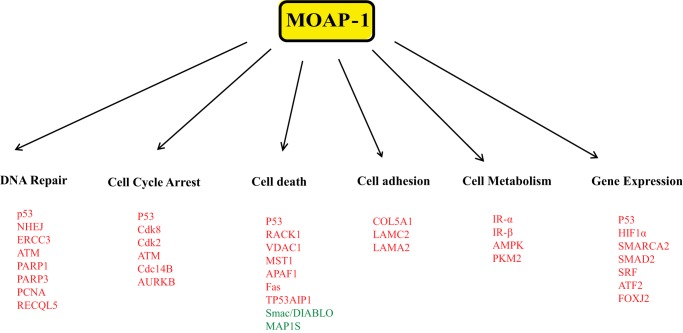
**Summary of genes modulated by the loss of MOAP-1.** *Green* names indicate down-regulation of expression; *red* names indicate up-regulation of expression.

Finally, modulators of gene expression were also differentially regulated in the presence of overexpressed MOAP-1. These include serum-response factor, E-26-like protein 1 (ELK1) transcription factor, and signal transducer and activator of transcription 3 (STAT3, validated in Fig. 10*C*). We were also able to validate the increase in both STAT3 and STAT5a, GNB2L1/RACK1, PTEN, and PARP (all validated in Fig. 10*C*) in xenograft-derived tumor lysates overexpressing MOAP-1, although the receptor for the STATs, Janus kinase 1 (JAK1), did not appear to be up-regulated (confirmed in Fig. 10*C*). Interestingly, the overexpression of MOAP-1 also resulted in modulation of key elements linked to metabolism and the Warburg effect (40–42). These include insulin receptor β, AMPK, and pyruvate kinase M2 (PKM2) (validated in Fig. 10*C*). MOAP-1 may thus have a role in modulating the appearance of biomarkers of the Warburg effect and in modulating metabolic pathways. Overall, our GWAS results suggest that MOAP-1 functions as more than just a pro-apoptotic protein and may be involved in numerous other aspects of homeostatic regulation in colon epithelial cells (Fig. 11).

## Discussion

Our current data support the role of MOAP-1 as a tumor suppressor involved in cell death, tubulin stability, and in several unexplored biological functions important for its ability to mediate growth suppression. MOAP-1 expression levels appear to be regulated by mRNA control, ubiquitination (Fig. 4) (3), and possibly by post-translational phosphorylation (Fig. 2*B*, *lanes 6–8*, slower migrating band). Analysis using Scan Site reveals the presence of several internal sequences that may be phosphorylated by aurora kinase A, glycogen synthase kinase 3β, Akt, polo-like kinase, protein kinase C isoforms μ, ϵ, and ζ, or the DNA damage kinase ATM, a kinase that can also phosphorylate the ATM site on RASSF1A (43). Evaluation of the role of these kinases is underway.

Immunostaining for MOAP-1 in several cancer cell lines illustrated that, under normal physiological conditions, the intracellular abundance of MOAP-1 is maintained at low levels as a result of its constitutive degradation by the ubiquitin-proteasome system (6) with APC/C^cdh1^ (7) similar to APC/C^cdc20^-directed ubiquitination of RASSF1A during mitosis (38, 44). It is possible that MOAP-1 ubiquitination is controlled in a cell cycle-specific manner by the APC/C^cdh1^ (7) pathway. This complex regulation may reflect the need to tightly control a pro-apoptotic molecule bridging both the extrinsic and intrinsic death receptor pathways.

Adding further evidence for its tumor suppressor function, our xenograft assays suggest that MOAP-1 can be additive to the tumor suppressor property of the RASSF1A (Fig. 7*B*) and that the pro-apoptotic function of MOAP-1 may govern the tumor suppressor function (Fig. 7, *C* and *D*). In addition, the important role of RASSF1A in modulating tubulin stability may be shared with its association with MOAP-1 to further strengthen the role of the RASSF1A/MOAP-1 tumor suppressor pathway. Utilizing splenocytes from a *Moap-1*^−/−^ mouse (courtesy of Victor Yu, National University, Singapore), we can observe a >2-fold increased cellularity in the *Moap-1*^−/−^ *versus* wild type mice to suggest importance in growth control.^5^ Furthermore, we have reconstituted *Moap-1* into *Moap-1*^−/−^ splenocytes and can observe reconstitution of cell death sensitivity upon intrinsic and extrinsic stimulation of *Moap-1*^−/−^ splenocytes.^5^ This strongly suggests a role for MOAP-1 in several modes of cell death and the mechanism of how it may control growth and behave as a tumor suppressor.

Our GWAS analysis clearly revealed elements that have been demonstrated to also influence or be influenced by RASSF1A, such as DNA damage control (ATM), Hippo pathway signaling (MST1) (12), cell cycle control (p53 and Aurora Kinases) (45), cell death (Fas and MAP1S) (37, 46), and cell signaling (Rap1) (47) to mention a few. The role for MOAP-1 in influencing the aforementioned elements is currently underway and will be informative. Our GWAS study also revealed the possible role for MOAP-1 in influencing pathways modulated by the Warburg effect. These include pyruvate kinase M2 (PKM2) (48) and AMPK (49) as described in Tables 2 and 3. Modulation of PKM2 and AMPK will influence how tumors survive in an environment of limited nutrient supply. We are currently exploring changes in metabolic parameters in the presence of overexpressed and unexpressed MOAP-1 expression.

A potentially interesting connection for MOAP-1-dependent tumor suppression is the link to DNA damage control by expression changes in PARP1 and PARP3 (key players involved in the detection and repair of DNA double strand breaks), ATM (a DNA damage checkpoint kinase), ATR-interacting protein, and the catalytic subunit of DNA-activated protein kinase (DNA-PK, a DNA damage sensor) (Tables 2 and 3). ATM can promote growth arrest and DNA repair through several different pathways involving hypoxia inducible factor 1α (HIF1α) (50), an element 3.7-fold increased in MOAP-1-overexpressing tumors (data not shown). Because defects in DNA repair pathways can lead to genomic instability and thereby promote carcinogenesis, it is imperative that we understand how MOAP-1 may influence DNA damage control. It is already known that RASSF1A can be modulated by ATM levels (43, 51) and associates with the nucleotide excision repair enzyme, XPA (52, 53). It will be interesting to explore how intimately connected MOAP-1 is to RASSF1A-modulated DNA damage control (or other biological processes) to better understand MOAP-1/RASSF1A-dependent and -independent functions.

Preliminary evidence does suggest that there is a strong positive correlation between RASSF1A and MOAP-1 expression in cancer cells. Both have now been demonstrated to be involved in TNF-R1-dependent cell death (8), tubulin stability (this study and Ref. 11), and tumor suppressor function (this study and Ref. 2). Furthermore, we can observe associations of both RASSF1A and MOAP-1 with the Toll receptor family of pattern both recognition receptors to affect NFκB activity (54).^5^ Thus, the RASSF1A/MOAP-1 molecular pathway may cooperate together to as a tumor suppressor pathway.

The results of our microarray (summarized in Fig. 11) reveals that MOAP-1 may be a tumor suppressor protein with multiple functions in physiology, influencing several molecular pathways connected to numerous aspects of biology. The MOAP-1 interactome needs to be better defined; how RASSF1A influences this interactome needs to be explored, and whether the loss or reduced expression of both RASSF1A and MOAP-1 is found in other diseases. Some of these questions are being currently explored as well as the links outlined in Fig. 11.

## Author Contributions

J. Law contributed to >60% of the data for the manuscript (including the writing and editing). M. S. contributed to Figs. 1*D* and 2*C*. A. Z. contributed to Figs. 1*E* and 2*D*. Y. W. contributed to early versions of Fig. 2*D* and to maintaining most of the cells in this work. L. L. contributed to the majority of Fig. 10. N. V. contributed to the editing of the manuscript and parts of Figs. 7 and 8. O. S. contributed to Fig. 6. K. F. contributed to parts of Fig. 5. J. Lim contributed to parts of Fig. 6. M. S. contributed to parts of Fig. 10. J. R. B. D. contributed reagents and time to parts of Fig. 10. C. T. T. provided MOAP-1 reagents for the study and contributed to the editing of the manuscript. Y.-C. S. contributed to the editing of the manuscript. V. C. Y. contributed to the editing of the manuscript and reagents for MOAP-1. J. M. contributed to Fig. 2, *A* and *B* (mainly the access to the breast cancer database used in Fig. 2). S. B. is the corresponding author overseeing this project and manuscript production.

## Footnotes

4 S. Baksh, O. Svystu, and K. Flood, unpublished observations.

5 S. Baksh and N. Volodko, unpublished observations.

## Footnotes

3 The abbreviations used are:

PARP: poly(ADP-ribose) polymerase
ATM: ataxia telangiectasia mutated
GWAS: genome-wide association study
MTT: 3-(4,5-dimethylthiazol-2-yl)-2,5-diphenyltetrazolium bromide
ER: estrogen receptor
PR: progesterone receptor
qPCR: quantitative PCR
PTEN: phosphatase and tensin homolog.
